# Synthetic peptides for the precise transportation of proteins of interests to selectable subcellular areas

**DOI:** 10.3389/fbioe.2023.1062769

**Published:** 2023-02-20

**Authors:** Junyi Song, Chuanyang Liu, Baoshan Li, Liangcheng Liu, Ling Zeng, Zonghuang Ye, Wenjian Wu, Lingyun Zhu, Biru Hu

**Affiliations:** Department of Biology and Chemistry, College of Science, National University of Defense Technology, Changsha, Hunan, China

**Keywords:** synthetic peptide, RfA1, preselected and spatiotemporal tunable, cyto/nucleoplasmic location, protein transportation

## Abstract

Proteins, as gifts from nature, provide structure, sequence, and function templates for designing biomaterials. As first reported here, one group of proteins called reflectins and derived peptides were found to present distinct intracellular distribution preferences. Taking their conserved motifs and flexible linkers as Lego bricks, a series of reflectin-derivates were designed and expressed in cells. The selective intracellular localization property leaned on an RMs (canonical conserved reflectin motifs)-replication-determined manner, suggesting that these linkers and motifs were constructional fragments and ready-to-use building blocks for synthetic design and construction. A precise spatiotemporal application demo was constructed in the work by integrating RL_Nto2_ (as one representative of a synthetic peptide derived from RfA1) into the Tet-on system to effectively transport cargo peptides into nuclei at selective time points. Further, the intracellular localization of RfA1 derivatives was spatiotemporally controllable with a CRY2/CIB1 system. At last, the functional homogeneities of either motifs or linkers were verified, which made them standardized building blocks for synthetic biology. In summary, the work provides a modularized, orthotropic, and well-characterized synthetic-peptide warehouse for precisely regulating the nucleocytoplasmic localization of proteins.

## 1 Introduction

Peptides and their derivatives are highly versatile structural and functional building blocks due to the richness of the amino acid arrangements and combinations available (Zelzer and Ulijn, 2010; Groß et al., 2016; Acar et al., 2017). On one hand, artificially designed peptides can generate various architectures [including fibers, tapes, tubes, sheets, and spheres (Acar et al., 2017)] *in vitro*, demonstrating the considerable potential for carrier-mediated drug delivery, tissue engineering, antimicrobial agents, imaging tools, energy storage, biomineralization, and membrane protein stabilization (Mandal et al., 2014). On the other hand, peptides and relative derivatives have been developed as effective navigation systems to selectively target organelles, e.g., endoplasmic reticulum (Field et al., 2015; Wang et al., 2019), mitochondria (Szeto et al., 2011; Jean et al., 2016), or nucleus (Beyer et al., 2015; Yumerefendi et al., 2015). Since the exact localization of proteins is required to fulfill their biological functions (Itzhak et al., 2016), transportation of functional proteins or peptides to orientated intracellular localization is a prerequisite to intensify their functions in application areas (Niopek et al., 2014; Guntas et al., 2015) or to study their mechanism in basic research fields (Drake et al., 2010; Slootweg et al., 2010). Bidirectional transportation of proteins in or out of nuclear membranes is such a dogmatic example. Inwards, proteins are translated into the cytoplasm, but many need to be transported into the nucleus to perform their functions (Christie et al., 2016). Outwards, RNA-protein complexes need to be dynamically exported from the nucleus into the cytoplasm (Grünwald et al., 2011; Niopek et al., 2016).

At this point, molecular tools to quantitatively regulate the entry and exit of target proteins into and out of the nucleus are of great value, which brings in various novel applications in synthetic and cell biology fields (Beyer et al., 2015; Niopek et al., 2016; Vogel et al., 2017).

Genetical construction of nuclear localization signal (NLS) sequences into cargo proteins is the most routine approach, which has successfully transported functional proteins (Beyer et al., 2015; Yumerefendi et al., 2015), genome-editing elements (Shi et al., 2019; Zhang et al., 2019), and transcriptional circuits (Khalil et al., 2012; Fonseca et al., 2019) into the nucleus. Similar but in the opposite direction, the utilization of NES (nuclear export signal) allows the translocation of molecular components out of the nucleus (Beyer et al., 2015; Lerner et al., 2018). Reorientated trafficking can further present light-responsive properties by embedding light-activated domains into NLS or NES (Engelke et al., 2014; Lerner et al., 2018; Allen et al., 2019). Besides, various NLS sequences have been used as delivery agents to enhance the cellular uptaking and nuclear targeting of plasmid DNA (Kim et al., 2012; Aied et al., 2013; Nematollahi et al., 2018), or other functional nano cargoes (Tammam et al., 2015; Yang et al., 2015; Yang et al., 2016). In this regard, the identification and engineering of intracellular guiding sequences from natural proteins will bring in prosperous advanced applications.

Reflectin proteins, exclusively expressed by Cephalopods (squid, octopus, and cuttlefish), are one group of unique functional proteins dominating the formation of biophotonic systems and manipulating structural coloration. Reflectins are highly enriched in periodically stacked lamellae called Bragg reflectors in iridocytes, which present iridescence by multilayer interference (Tao et al., 2010; DeMartini et al., 2013). While reflectins in leucophores are located in granular vesicles, responsible for producing bright white by unselectively reflecting all incident light (Williams et al., 2019). Intricate reflectin-based biophotonic systems have already inspired the development of various next-generation tunable photonic, though the underlying biological mechanism remains elusive (Qin et al., 2013; Phan et al., 2015; Dennis et al., 2017) and electronic platforms and devices (Ordinario et al., 2014; Yu et al., 2014; Phan et al., 2016). More significantly, groundbreaking attempts have been made to use reflectins as molecular tools to modify mammalian cell functions. Recently, Chatterjee et al. (2020) and Ogawa et al. (2020) expressed reflectins in the human embryonic kidney (HEK) 293 cells. After the formation of phase-separated aggregates, engineered human cells obtained an outstanding new feature, tunable optical properties. This is a milestone in discovering novel biosynthetic tools and endows mammalian cells with new features (Tang et al., 2020).

Based on similar considerations, HEK-293T cells were employed as a platform to explore the biological mechanism of reflectins and their functional potentials. Other than Atrouli and Junko’s brilliant findings (Chatterjee et al., 2020; Ogawa et al., 2020), our previous work was devoted to understanding the extensive interaction of reflectins with other cellular components (e.g., cytoskeleton) and explaining the formation of indelicate biophotonic structures fabricated by reflectins (Song et al., 2022). Reflectin A1, A2, B1, and C were found to present distinguished cyto-/nucleoplasmic localization preferences during this process. As natural block copolymers composed of positively charged polyelectrolyte linker regions (reflectin linkers, RLs) interspersed with highly conserved polyampholyte segments (reflectin motifs, RMs) (Levenson et al., 2019; Song et al., 2020), several RLs and RMs are speculated to dominate the subcellular distribution of different reflectins.

Hence, taking RLs and RMs as well-prepared and ready-to-use building blocks, they verify the hypothesis and designed a novel guiding system based on programmable RfA1 sequences, which could precisely transport peptides cargos to selective intracellular regions (nucleoplasm and cytoplasm).

As the first step, native reflectins RfA1, RfA2, RfB1, and RfC were introduced into HEK-293T cells and found to be preferentially enriched in nuclei or cytoplasm. Considering their sequence differences, the repetition of conserved motifs was likely to be in charge of selective intracellular localization. RfA1 and its derivatives were designed and engineered into cells to confirm it. RL_Nto1_, RL_Nto2,_ and RL_Nto3_ were found to transport GFP (as a cargo molecule) into the nucleus, while RL_Nto5_ caused a prominent cytoplasmic enrichment of GFP outside the nucleus. This strict intracellular localization of RfA1 derivatives confirmed the motif-repetition-dependent hypothesis and suggested them as editable guiding tags to transport molecular cargoes to selective regions. Subsequently, the precise nuclear enrichment was then temporally regulated with the administration of doxycycline by integrating the Tet-On system (T Das et al., 2016; Zhou et al., 2006) with RL_Nto2_. In this case, Tet-On components worked as the launch button, while RfA1-derived sequences were guided missiles that carried molecular cargos to prefixed targets. At last, genes of two recombinationally designed peptides RM_N_ + RM_1_*5 and RM_1_*3 + RL_2_*2 were synthesized to verify the functional homogeneity of RMs and RLs during subcellular localization. Subtle differences among motifs or linkers were eliminated for these two peptides. The distribution of these two recombinational peptides was exactly similar to comparable RfA1 derivatives in this assay, indicating that these peptide building blocks could be unified and standardized.

Briefly, a series of building blocks were identified from reflectin amino acid sequences by this work. Reorganization of these building blocks led to an accurate cytoplasmic or nucleoplasmic enrichment of ligated molecular cargos (e.g., GFP), which was quantified by repetitions between RMs and RLs. Combined with other synthetic biology-based tools, this programmable RfA1-derived strategy can be further upgraded as a spatiotemporal controllable toolkit to realize precise intracellular delivery.

## 2 Results

### 2.1 Subcellular localization of reflectin proteins and deconstruction of RfA1 sequence

**FIGURE 1:**
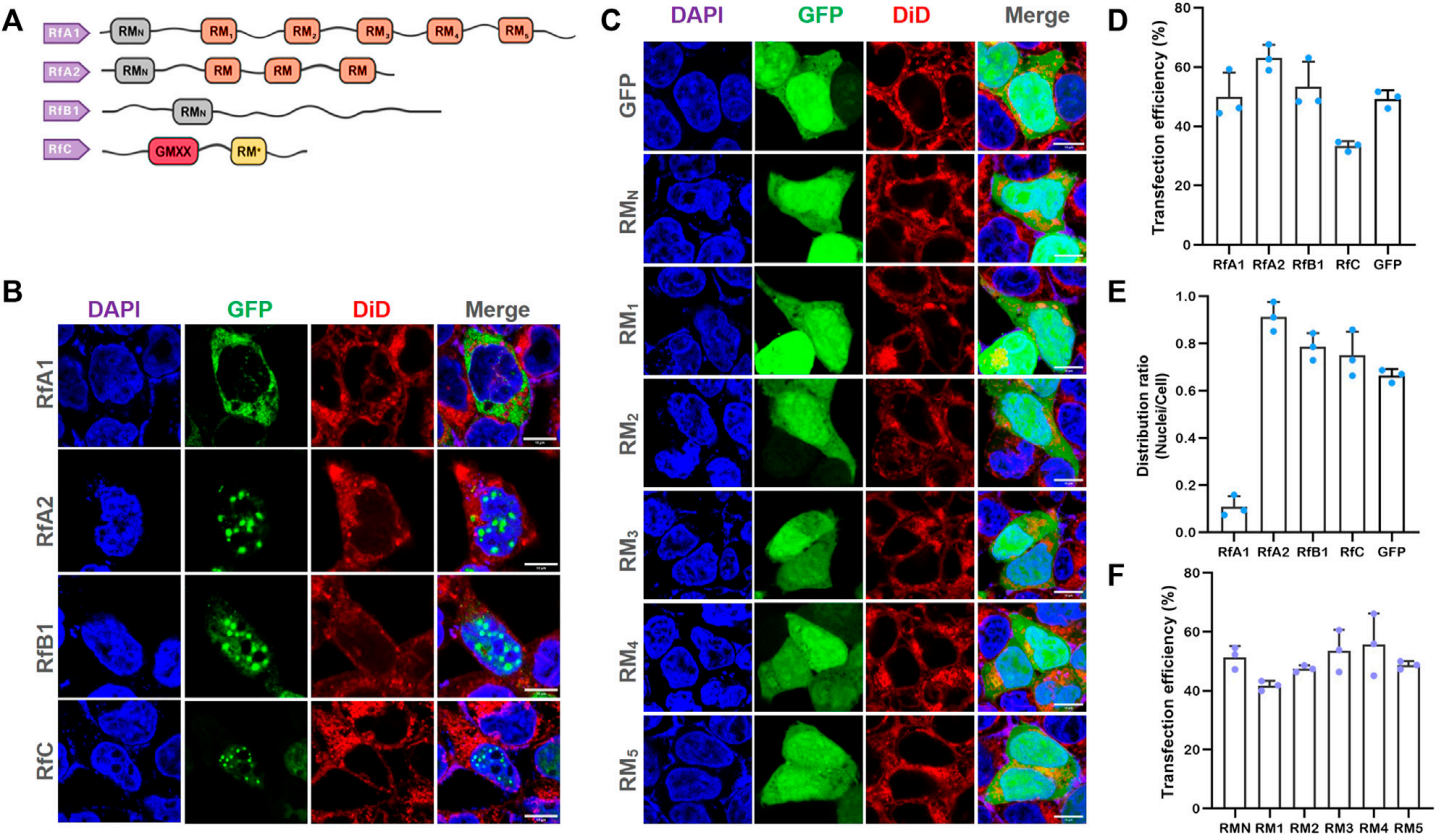
Selective Intracellular Localization of Reflectins and Free-Distribution of Single Motifs. **(A)** Schematics of reflectin proteins sequences. Conserved Reflectin Motifs (RM_N_ and repeated RM_1-5_) are designated by boxes, while reflectin linkers (RLs) are lines. **(B,C)** Fluorescence microscopy images of transfected HEK-293T cells stained with DAPI and DiD. While reflectins and variants are visualized by tandem EGFP, Scalebar = 10 μm. **(D)** Cell number statistics and quantification of transfection efficiencies of four reflectins and GFP. **(E)** Distribution ratio statistics of fluorescent intensity in transfected cells and their nuclei. **(F)** Cell number statistics and quantification of transfection efficiencies of five single RMs. Data are presented as mean values ±SD for *n* = 3 independent experiments.

Two types of patterned ∼25-amino-acid methionine-rich motifs are reported in reflectin sequence: N-terminal motif (RM_N_) [MEPMSRM(T/S)- MDF(H/Q)GR(Y/L)(I/M)DS(M/Q)(G/D)R(I/M)VDP (R/G)] and a series of conserved reflectin motifs (RMs) [M/FD(X)_5_MD(X)_5_MDX_3/4_] (Crookes et al., 2004) (see Figure 1A and Supplementary Figure S1). The N-terminal region is more evolutionarily conserved across species (*Doryteuthis opalescens*, *Doryteuthis pealeii*, *Loligo forbesii*, *Sepia officinalis*, *Euprymna scolopes*, and *Octopus bimaculoides*) and reflectin isoforms (23 in 27 kinds of the most known reflectins) than canonical RM_N_ (Izumi et al., 2010). While almost all “X” sites are populated largely by one specific residue with minor alternative residues usually represented in only one or a few reflectin motifs in the entire known library. Specifically, RfC is the shortest reflectin containing a GMXX motif and RM*. The GMXX motif is a unique region of increased overall hydrophobicity composed of a four amino acid repeat, where ‘X’ represents less conserved locations within the repeat. Asterisk-marked RM* of RfC contains substantial deviations in the sequence not observed in any other reflectin motifs (Levenson et al., 2017). Supplementary Figure S1 shows reflectins and their RM_N_ and RMs.

At present, studies are mostly focused on their self-assembly properties *in vitro* (DeMartini et al., 2015; Levenson et al., 2016; Levenson et al., 2019). The dynamic reflectin assembly properties have already inspired the development of various next-generation tunable photonic (Qin et al., 2013; Phan et al., 2015; Dennis et al., 2017) and electronic platforms and devices (Ordinario et al., 2014; Yu et al., 2014; Phan et al., 2016).

Four reflectin proteins were constructed into pEGFP-C1 vectors and transfected into HEK 293T cells to further investigate their intracellular characteristics. Compared with cells transfected by no-load pEGFP-C1, all these four kinds of reflectins tend to form protein condensates or spherical droplets in cells (see Supplementary Figure S2 for large area immunofluorescence images and Supplementary Figure S3 for cell viability tested by CCK-8 kits). The formation of proteinaceous condensates is consistent with speculation that reflectins are the potential intrinsically disordered protein to execute phase separation (Levenson et al., 2019).

More significantly, RfA1 condensates were exclusively distributed in the cytoplasm, while RfA2, RfB1, and RfC droplets were highly enriched in nuclei (see Figures 1B, E). Since amino acid composition lays the foundation of a protein structure and function, the similarities and differences among reflectins are also determined by their primary structure. The reason why they are sorted into one protein family is reflectin motifs (RMs) that they share. Meanwhile, the most significant difference among reflectins is also the number of RMs repetitions. As suggested by Morse and coworkers, these canonical reflectin motifs (RMs) could be structural or functional elements of reflectins (Levenson et al., 2016). Seeing from this point, cytoplasm-retained RfA1 contains the largest numbers of reflectin motifs, while nucleoplasm-enriched reflectins contain less. Hence, the work cloned the six RMs of RfA1 (RM_N_, and RM_1,2,3,4,5,_ primers in Supplementary Table S1) and introduced them into cells to explore the role of conserved RMs during the protein condensation and selective localization. Results showed that all individual RMs distributed freely in both cytoplasm and nucleoplasm, with no difference compared to GFP alone expressed in cells (see Figure 1C). Therefore, the cytoplasmic enrichment of reflectins other than conserved amino acid composition should be driven by its segmented sequence structure. Transfection efficiencies were calculated and shown in Figures 1D/F.

### 2.2 Reconstruction of block amino acid sequence

**FIGURE 2:**
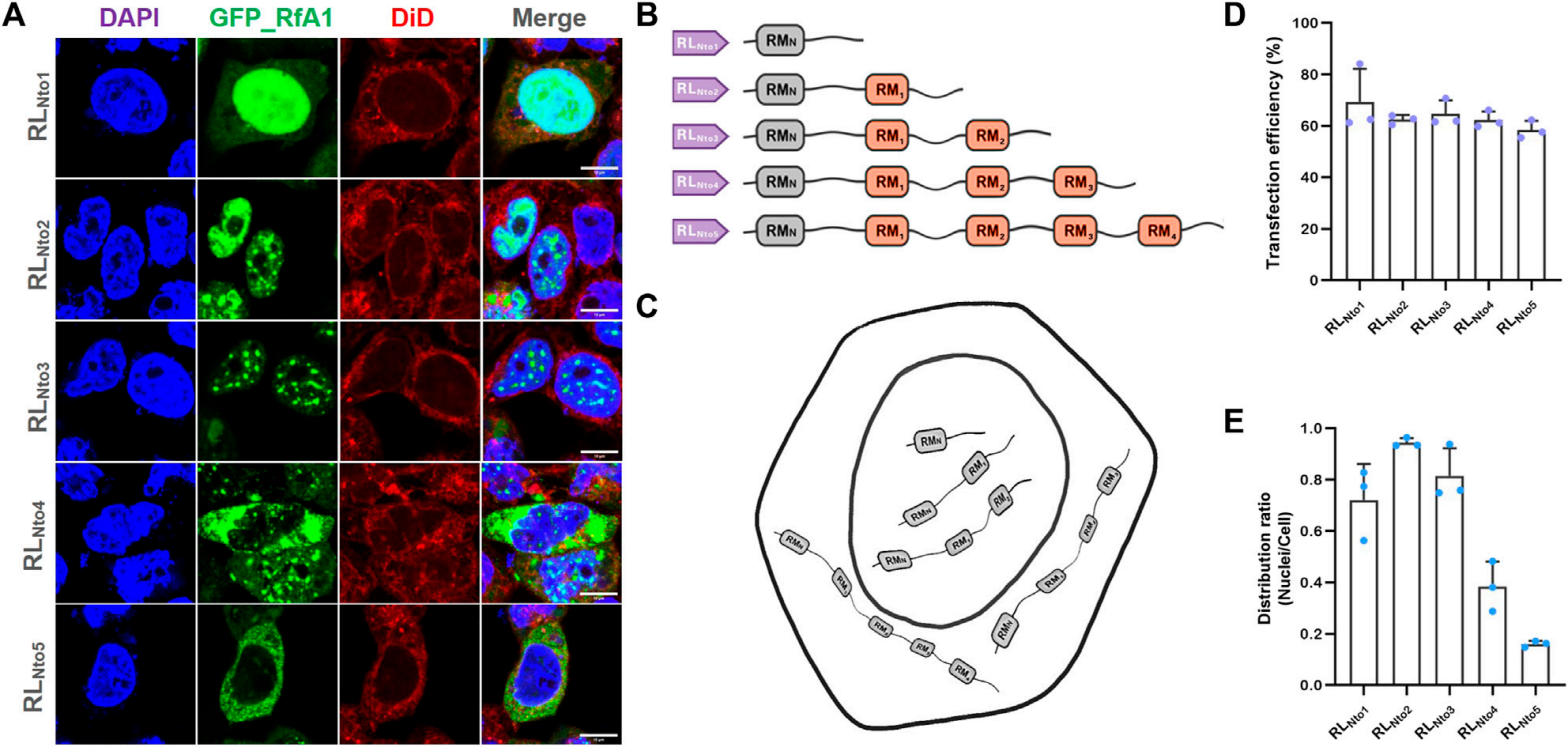
Recurrence of phase separation and cyto-/nucleo-localization preferences of RfA1-derived peptides in fixed HEK-293T cells. **(A)** Nuclei and membrane are stained with DAPI (blue) and DiD (red), and RfA1-derived peptides are indicated by tandem EGFP (green). Scalebars = 10 μm. **(B)** Schematics of RfA1 derivatives. **(C)** Illustration of subcellular localization of RL_Nto1_, RL_Nto2_, RL_Nto3_, RL_Nto4,_ and RL_Nto5_. **(D)** Cell number statistics and quantification of transfection efficiencies of RL_Ntox_ (x = 1, 2, 3, 4, and 5). **(E)** Distribution ratio statistics of fluorescent intensity in transfected cells and their nuclei (see Supplementary Figure S4 for large-area immunofluorescence images and Supplementary Figure S3 for Cell viability tested by CCK-8 kits). Data are presented as mean values ±SD for *n* = 3 independent experiments.

Five pairs of primers were designed to clone the DNA sequences from RfA1 genes (see Supplementary Table S2 for primers) and gradually extend peptide sequences and restore their segmented structure. PCR products responsible for coding RL_Nto1_, RL_Nto2_, RL_Nto3_, RL_Nto4,_ and RL_Nto5_ are subsequently ligated to vector pEGFP-C1. The common characteristic for cells expressed with RL_Nto1_, RL_Nto2_, and RL_Nto3_ is their enrichment in nuclei (see Figures 2A, E). It is different from the cytoplasmic-localization-preference of RfA1 or free-distribution of single RMs, but quite similar to simpler reflectins (RfA2, RfB1, and RfC). Besides, RL_Nto2_ and RL_Nto3_ are enriched in cytoplasm and phased out from the crowded cellular milieu (see Figures 2A, E). As sharp contrasts, longer RL_Nto4_ and RL_Nto5_ start to be excluded from nuclei and form condensates in the cytoplasm (see Figures 2A, E), which extremely resembles RfA1(see Figures 1B, E).

Guan et al. (2017) reported that reflectin motifs may be traced to a 24-bp transposon-like DNA fragment from symbiotic bioluminescent bacterium *Vibrio fischeri* in 2017. Afterward, million years of self-replication and translocation of that transposon leads to the formation of reflectin motifs and the prosperous reflectin family. Here, the repetition numbers of RMs (as basic units) accurately determine the different properties (e.g., intracellular localization) among RfA1, A2, B1, and C. Being a subordinate element and evolutionary origin, the 24-bp transposon-like DNA fragment is expected to be the root source of reflectins diversification, which makes our finding a strong clue to support Guan’s evolution hypothesis.

On the other hand, if GFP is taken as a molecular cargo, reflectin derivatives can be regarded as intelligent vehicles to transport cargoes to pre-selected destinations (cytoplasm or nucleoplasm). Based on this consideration, we tested the application potentials of RfA1 variants as synthetic biology tools. As the shortest peptide strictly targets nuclei, RL_Nto2_ was used in the following studies and regarded as a guiding tag, with transfection conditions optimized by dose-dependent and time-scale preliminary assays (see Supplementary Figure S5).

### 2.3 Doxycycline-induced Tet-On system integrated with RL_Nto2_

**FIGURE 3:**
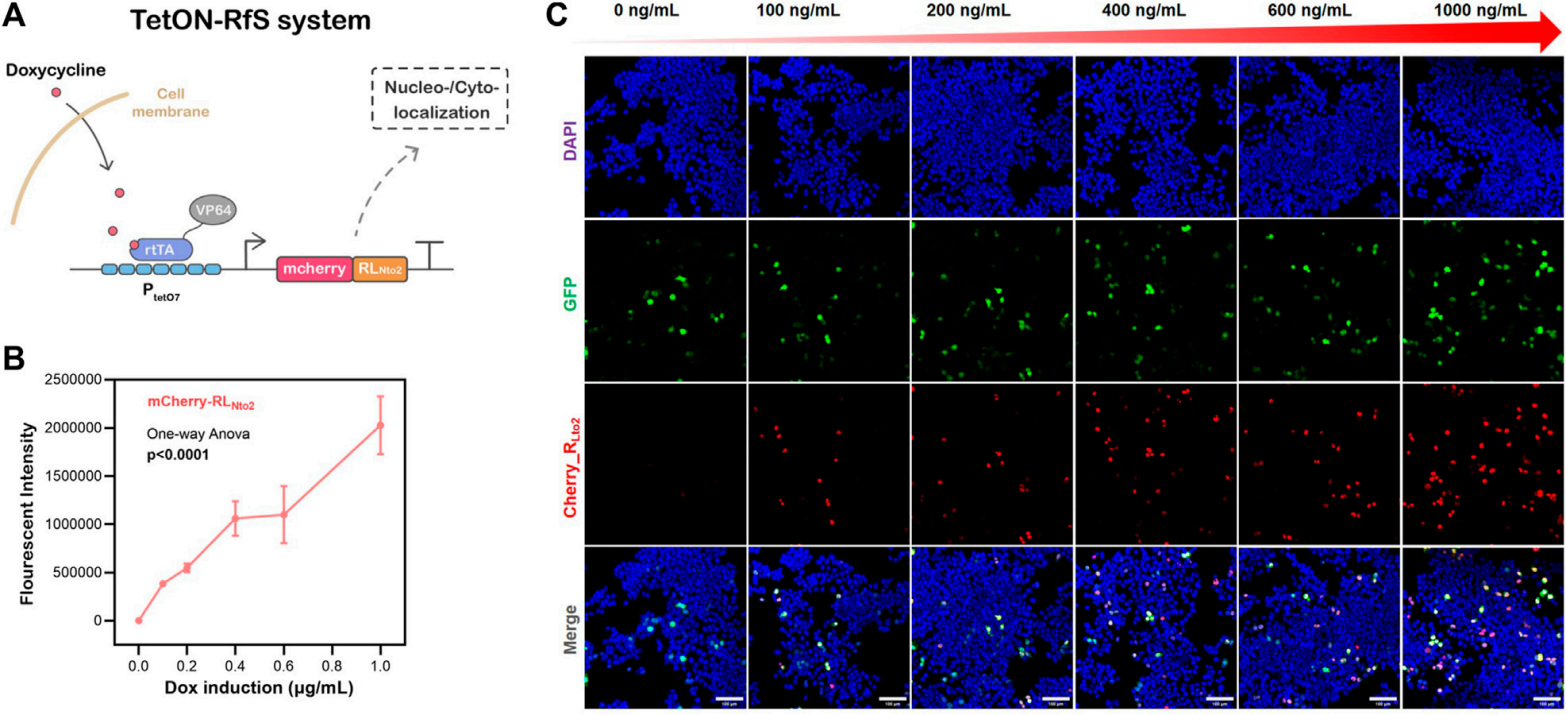
Dose-dependent nuclei-targeted Tet-On-RL_Nto2_ system mediated by doxycycline. **(A)** schematic diagram of the Tet-On-Rfs system. **(B)** Total Intensity, which represents the gross expression of mcherry_RL_Nto2._ Data are presented as mean values ±SD for *n* = 3 independent experiments. **(C)** Activation of the Tet-On-Rfs system. Transfected cells are indicated by GFP (green), and the localization of RL_Nto2_ is labeled by tandem mCherry (Red); Scalebars = 100 μm.

RL_Nto2_ is constructed into Tet-On plasmids that can be easily switched on or off by doxycycline (dox) to exhibit its application potential as a synthetic biology component (see Figure 3A). When cell confluence reaches ∼30%, transfection is conducted according to Lipofectamine 3,000 protocol. Transfection efficiency is checked by fluorescent images after 24 h. Cells are then treated with concentration-gradient dox and cultivated for another 24 h. Afterward, the nuclei-targeted expression of RL_Nto2_ is observed (see Figures 3B, C and Supplementary Figure S6) by confocal microscope. The expression level of RL_Nto2_ is enhanced synchronously with the dox concentration gradient (see Figure 3C). This implies the successful activation of this Tet-On system and its controllability based on the dox dose-dependent behavior.

Foreseeable, if reporter gene *mCherry* was replaced by other functional or therapeutical peptides, the Tet-On-Rfs system can precisely transport the molecular cargos into nuclei to amplify their biological effects. Besides, if certain components prefer to fulfill their functions in the cytoplasm, RL_Nto2_ can be replaced by RL_Nto4_ or RL_Nto5_. At this point, the programmable RfA1 sequences provide an editable and selectable engineered toolkit to precisely transport proteins or peptide cargoes to the preselected subcellular area.

### 2.4 Design of a blue-light-controlled subcellular enrichment system

Based on Figure 2, RfA1-derived peptides occupying more than 3 RM motifs tend to stay in the cytoplasm. Otherwise, shorter peptides are inclined to be enriched in nucleoplasm. Perspectively, peptides RL_Nto2_ can travel across nuclear membranes, while its dimer or analog may be resisted. Hence, CoH2/DocS domains were introduced for their stable and high-affinity interaction to verify this speculation (Li et al., 2020; Yu et al., 2020).

**FIGURE 4:**
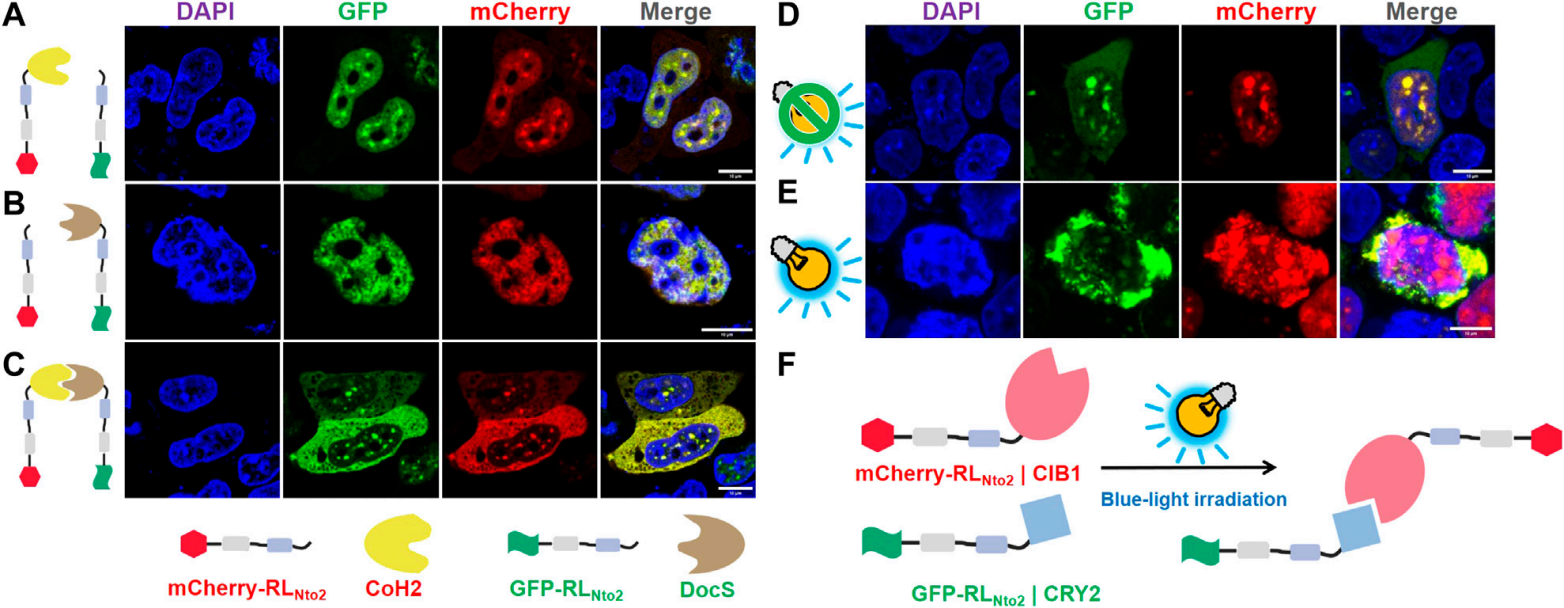
Restoration of repetition-level dependent cytoplasmic enrichment. **(A)** Confocal observation of HEK-29ET cells transfected with pCDNA3.1-mCherry-RL_Nto2_-CoH2 and pCDNA3.1-GFP-RL_Nto2_. **(B)** Confocal observation of HEK-29ET cells transfected with pCDNA3.1-mCherry-RL_Nto2_ and pCDNA3.1-GFP-RL_Nto2_-DocS. **(C)** Confocal observation of HEK-29ET cells transfected with pCDNA3.1-mCherry-RL_Nto2_-CoH2 and pCDNA3.1-GFP-RL_Nto2_-DocS. **(D,E)** Confocal observation of HEK-29ET cells transfected with CDNA3.1-mCherry-RL_Nto2_-CIB1 and pCDNA3.1-GFP-RL_Nto2_-CRY2, treated with or without blue light, respectively. **(F)** Illustration for the blue-light induced interaction between recombinant mCherry-RL_Nto2_-CIB1 and GFP-RL_Nto2_-CRY2. Scalebars = 10 μm.

Recombined proteins mCherry-RL_Nto2_-CoH2 and GFP-RL_Nto2_ can pass through nuclear membranes and get into nuclei (see Figure 4A). Since these two proteins share the same transmembrane mechanism, they induce similar molecular responses in nuclei and co-localized to each other. Similarly, co-expression of mCherry-RL_Nto2_ and GFP-RL_Nto2_-DocS leads to the same phenomenon (See Figure 4B). However, when CoH2 and DocS domains are fused to mCherry-RL_Nto2_ and GFP-RL_Nto2_, respectively, most of the fluorescence signal is retained in the cytoplasm (see Figure 4C). The results suggest that the repetition-dependent nucleocytoplasmic localization preference of RfA1-derived peptides can be adjusted by molecular splicing. Inspired by this, the CRY2–CIB1 system is then employed to generate photoactivatable subcellular localization. mCherry-RL_Nto2_-CIB1 and GFP-RL_Nto2_-CRY2 without blue-light stimulus tend to be enriched in nuclei and co-localize with each other (Figure 4D). Contrastly, the green fluorescent signal of GFP-RL_Nto2_-CRY2 in a fraction of cells was prevented from entering the nuclei upon blue-light irradiation (Figure 4E). In this case, blue-light induced interaction between CIB1 and CRY2 elevates the RMs repetition level because RL_Nto2_ turned into dimer analog RL_Nto2_-Pairs- RL_Nto2_ and leads to their retention in cytoplasm (Figure 4F) .

### 2.5 Verification of standardization of reflectin-derived building blocks

**FIGURE 5:**
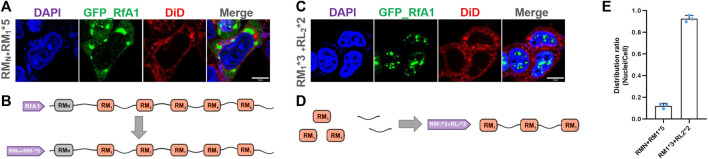
Homogeneity/standardization of reflectin-derived building blocks. **(A,C)** Fluorescence images of fixed HEK-293T transfected with pEGFP-C1-(RM_N_ + RM_1_*5) and pEGFP-C1-(RM_1_*3 + RL_2_*2), respectively. The nucleus and cytomembrane are stained with DAPI and DiD. **(B,D)** Schematics of RM_N_ + RM_1_*5 and RM_1_*3 + RL_2_*2 sequences. **(E)** Distribution ratio statistics of fluorescent intensity in transfected cells and their nuclei. Data are presented as mean values ±SD for *n* = 3 independent experiments.

Building blocks used for synthetic biology should better be standardized and modalized. Although the RMs in reflectins are highly canonical and conserved, there are still subtle composition differences among them (Crookes et al., 2004; Izumi et al., 2010; Levenson et al., 2017). We replaced all RM_1∼5_ in RfA1 with a unified RM_1_ to eliminate this subtle composition discrepancy (see Supplementary Table S3 for sequence information and Figure 5B for sketches of artificial peptides). Being similar to the original RfA1, RM_N_ + RM_1_*5 is also observed to be highly enriched in cytoplasm (Figures 5A, E). This unification process (RM_1∼5_ into RM_1_) will not change proteins’ properties at least for intracellular localization preferences. Moreover, one recombinational peptide RM_1_*3 + RL_2_*2 is designed (see Supplementary Table S3 for sequence information; Figure 5D for the sketches of artificial peptides). It is transported and enriched in nuclei Figure 5C, which is similar to RfA1-derived analog RL_Nto2_ (Supplementary Figure S6C, E). Hence, building blocks or functional components derived from reflectin amino acid sequences can be standardized without losing their intracellular localization preferences, which favors their application in synthetic biology.

## 3 Discussion

As essential biomacromolecules, the exact localization of proteins is required for organelles to work correctly (Itzhak et al., 2016). Proteins are translated into cytoplasm, but many need to be transported into nuclei to perform their functions (Christie et al., 2016). On the other hand, the dynamic export of RNA-protein complex out from nuclei is also a central but not fully understood process for molecular biology (Grünwald et al., 2011; Niopek et al., 2016). Hence, molecular tools, which qualitatively and quantitatively regulate the entry and exit of target proteins into and out of nuclei, bring in various novel applications in synthetic and cell biology (Beyer et al., 2015; Niopek et al., 2016; Vogel et al., 2017).

The work found the different intracellular localization preferences among reflectin proteins. Based on this property, a series of synthetic peptides were designed to precisely target preselected destinations. Moreover, the selectable enrichment of reflectin-derived peptides and conjugated molecular cargoes can further be temporal-/spatial-tunable upon chemical or light stimuli by integrating with other well-established cell biology tools, demonstrating their potential as biosynthetic elements.

Being curious about their intracellular functions and properties, genes of four native reflectins (RfA1, RfA2, RfB1, and RfC) were introduced into HKE-293T cells. Interestingly, the localization preferences of reflectins were distinguishable: RfA1 was exclusively located in the cytoplasm, while RfA2, RfB1, and RfC were highly enriched in the nucleoplasm. Being natural block copolymers, reflectins are composed of positively charged polyelectrolyte linker regions (reflectin linkers, RLs) interspersed with highly conserved polyampholyte segments (reflectin motifs, RMs). The most significant difference among reflectins is the number and localizations of RMs.

Hence, reflectin sequences provide programmable building blocks to guide cargo molecules and achieve selective subcellular localization from a biosynthetic application perspective. RfA1 was taken as the initial template by cutting RMs off one by one from the RfA1 amino acid sequence *via* gene engineering. Longer RfA1 derivatives with more RMs repetitions tended to stay in the cytoplasm, while shorter RfA1 truncates started to enter nuclei. Moreover, we integrated the RfA1-derived guiding peptides with the Tet-On system. Tet-On elements worked as a trigger in this demo, which allowed precise activation at the scheduled time point under the regulation of doxycycline. While RfA1 derivatives RL_Nto2_ worked as precision-guided systems, which carry the molecule cargo (e.g., mcherry) into nuclei.

The repetition-dependent nucleocytoplasmic localization preference of RfA1-derived peptides could be adjusted by molecular splicing. Increased RMs repetition level prevented dimer-analog RL_Nto2_-CoH2/DocS-RL_Nto2_ from entering nuclei and made these combined proteins stay in the cytoplasm by integrating nucleoplasmic-preferential RL_Nto2_ with high-affinity interaction pair CoH2/DocS. The subcellular localization preference of RfA1-derived peptides was even controllable by replacing the CoH2/DocS system with a blue-light regulated CRY2/CIB1 system. These results strongly implied the expansibility of the RfA1-derived molecular toolkit by integrating them into other synthetic biology systems. However, the optimal conditions to precisely regulate this blue-light-controlled intracellular distribution system are not adequately acquired at this stage, including plasmids ratio, light intensity, and treatment duration. Since the blue-light receptor cryptochrome underwent oligomerization when transducing blue-light signals after irradiation (Ma et al., 2020), the blue-light induced retention of RL_Nto2_-CRY2/CIB1-RL_Nto2_ in the cytoplasm might be more complex and the underlying mechanism was not fully understood.

At last, the functional homogeneity of RMs and RLs was verified by replacing RM_2,3,4,5_ in RfA1 with unified RM_1_ or recombinationally designing an artificial peptide “RM_1_*3 + RL_2_*2.” Guiding sequences derived from the RfA1 amino acid sequence can be modified as unified and standardized building blocks for cyto- or nucleo-targeting. The RfA1-derived strategy and standardized building blocks can be further programmed and developed as versatile and spatiotemporal controllable toolkits by combining them with other responsive synthetic biology components.

## 4 Methods

### 4.1 Construction of recombinant pEGFP-C1 vectors

The nucleotide sequences of D. (Loligo) pealeii reflectin A1 (RfA1) (Genbank: ACZ57764.1), D. (Loligo) pealeii reflectin A2 (RfA2) (Genbank: ACZ57765.1), D. (Loligo) pealeii reflectin B1 (RfB1) (Genbank: ACZ57766) and D. (Loligo) Opalescens reflectin C (Genbank: AIN36559.1) were optimized for human-cell expression. Then, they were synthesized and sequencing-identified by Sangon Biotech^®^ (Shanghai, China) Primers (F-GAATTCTATGAATAGATATTTGAATAGACA; R-GGATCCATACATATGATAATCATAATA ATTT) were designed to introduce EcoR I and BamH I cutting sites, so the modified RfA1 CDS could be constructed into pEGFP-C1 *via* a standard restriction enzyme cloning process. As for truncated RfA1 derivatives, six pairs of primers were coupled. If RMN-F and RM3-R primers were selected, then a nucleotide sequence responsible for coding of RMN-RL1-RM1-RL2-RM2-RL3-RM3 (simplified as RMNto3 in the work) was obtained after PCR. Meanwhile, 5′GCA​TGG​ACG​AGC​TGT​ACA​AG 3′ and 5′ TTATGATCAG- TTATCTAGAT 3’ were added to those F-primers and R-primers separately during primers synthesis, which enabled sequences to be ligated to pEGFP-C1 by Ready-to-Use Seamless Cloning Kit from Sangon Biotech^®^ (Shanghai, China).

### 4.2 Growth and transfection of human cells

HEK-293T cells (ATCC^®^, CRL-3216TM) were cultured on plastic dishes in Dulbecco’s Modified Eagle Medium (DMEM, GibcoTM) supplemented with 10% fetal bovine serum (FBS, GibcoTM) at 37°C and under 5% CO_2_. Cells were seeded at ∼33% of the confluent density for the glass bottom dishes from Cellvis (California, United States) 1 day before transfection, and grown for another 24 h. Then transfection mixtures containing Lipofectamine 3,000 (Ther-mo Scientific) and recombinant vectors were added to the medium and incubated for ∼16–∼24 h. 1 × 10^4^ cells were seeded into each hole of 96-well plates 1 day before transfection for CCK-8 tests. Then they were transfected with recombinant vectors and incubated for another 24 h. After that, 10-μL CCK-8 solutions were added into wells for a ∼2- to ∼4-h chromogenic reaction. OD450 was detected by Multiskan FC (Thermo Scientific).

### 4.3 Fluorescence microscopy of stained cells

Transfected HEK-293T cells grown in Cellvis plastic dishes were firstly fixed with 4% paraformaldehyde at room temperatures for 30 min and then stained with DiD (diluted in 0.5% Triton X-100 PBS) for ∼30 min after PBS rinses. Fixed cells were embedded in DAPI-Fluoromount (Beyotime, Shanghai, China) after washing off the fluorescent dye with PBS, and characterized with a Leica TCS SP8 imaging system in fluorescence imaging mode. 405 Diode laser was used for DAPI detection; 488 Argon was used for GFP detection; DPSS 561 was used for mcherry detection; HeNeB 633 was used for DiD detection. The resulting images were analyzed with ImageJ (Java 1.8.0_172/1.52b) (Schindelin et al., 2012).

## Data Availability

The datasets presented in this study can be found in online repositories. The names of the repository/repositories and accession number(s) can be found in the article/Supplementary Material.
