# Supplementary material for: Synthetic peptides for the precise transportation of proteins of interests to selectable subcellular areas
Source: Front Bioeng Biotechnol. 2023 Feb 20;11:1062769. doi: 10.3389/fbioe.2023.1062769 (PMC9986269; doi:10.3389/fbioe.2023.1062769)
Supplement: Supplementary file 2 [file DataSheet1.docx]

**Supporting files**


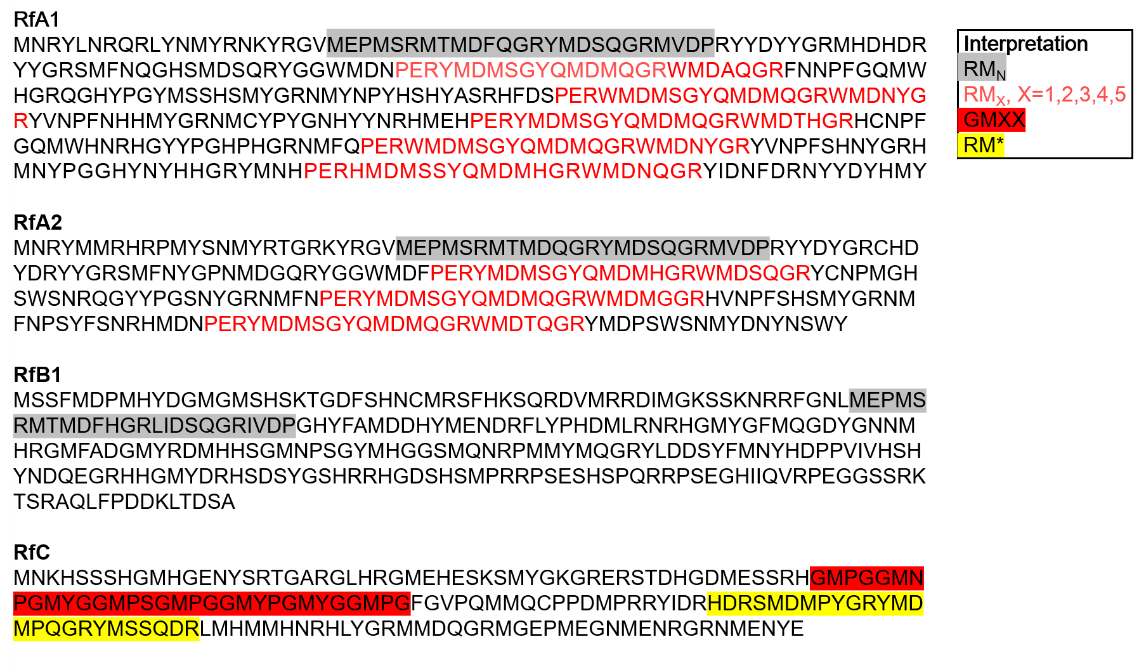


**Figure. S1** Sequences of reflectins, with motifs makred with different colors.


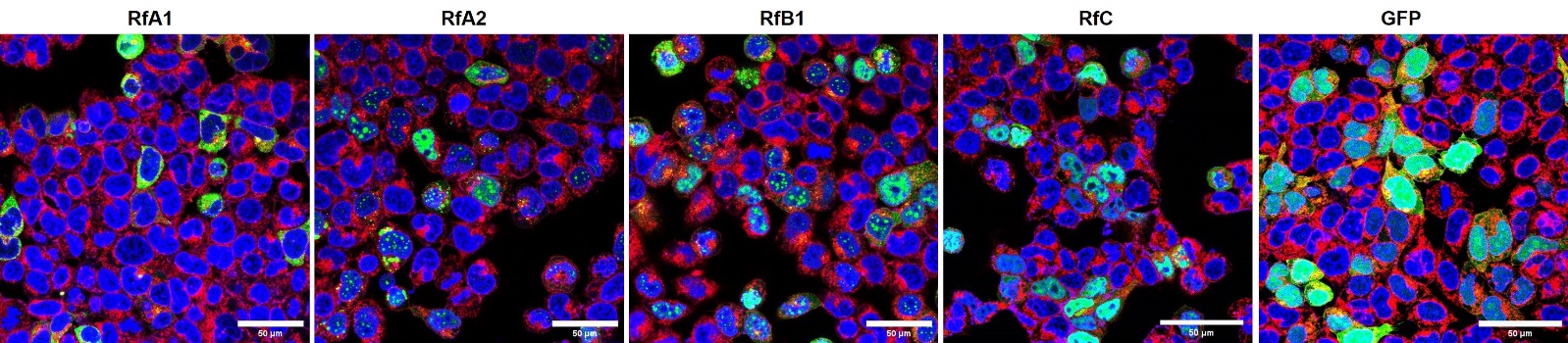


**Figure. S2** Large area immunofluorescence images of reflectins and GFP-only control.


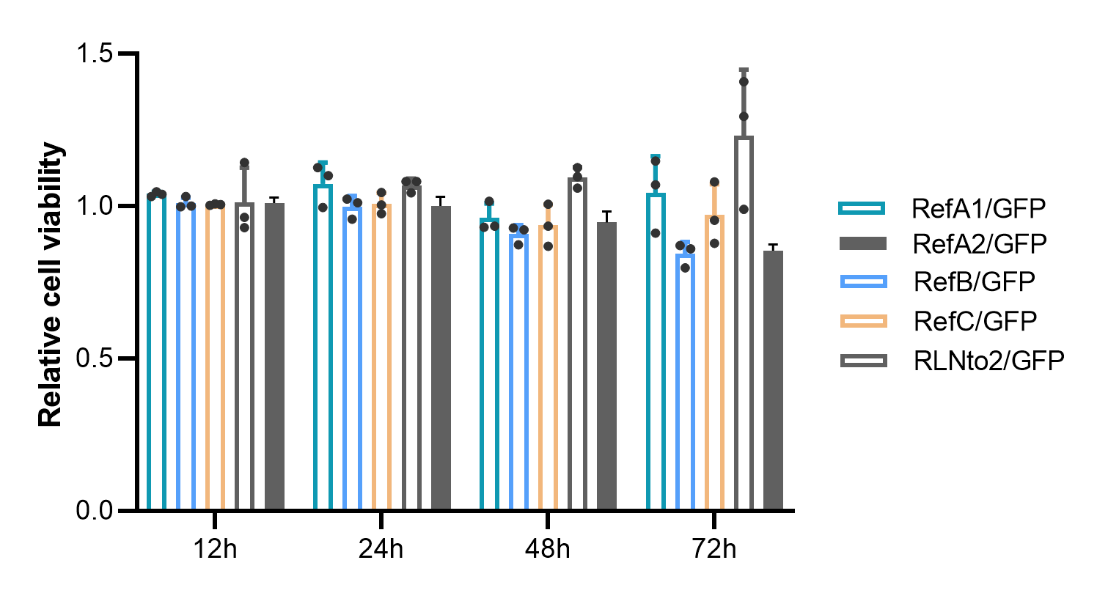


**Figure. S3** Cell viability tested by CCK-8 kits, after transfection of recombinant plasmids for 12hr, 24hr, 48hr and 72 hr.


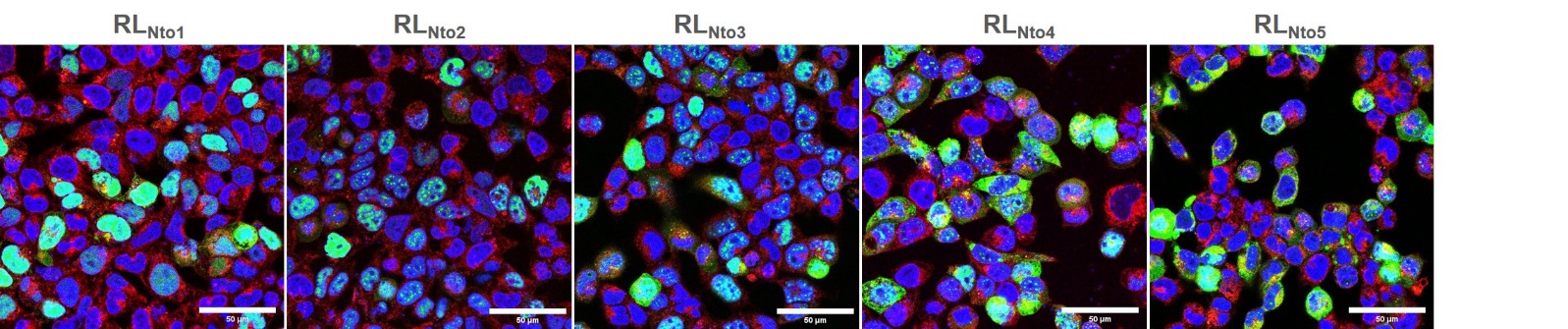


**Figure. S4** Large area immunofluorescence images of RfA1 derivates.


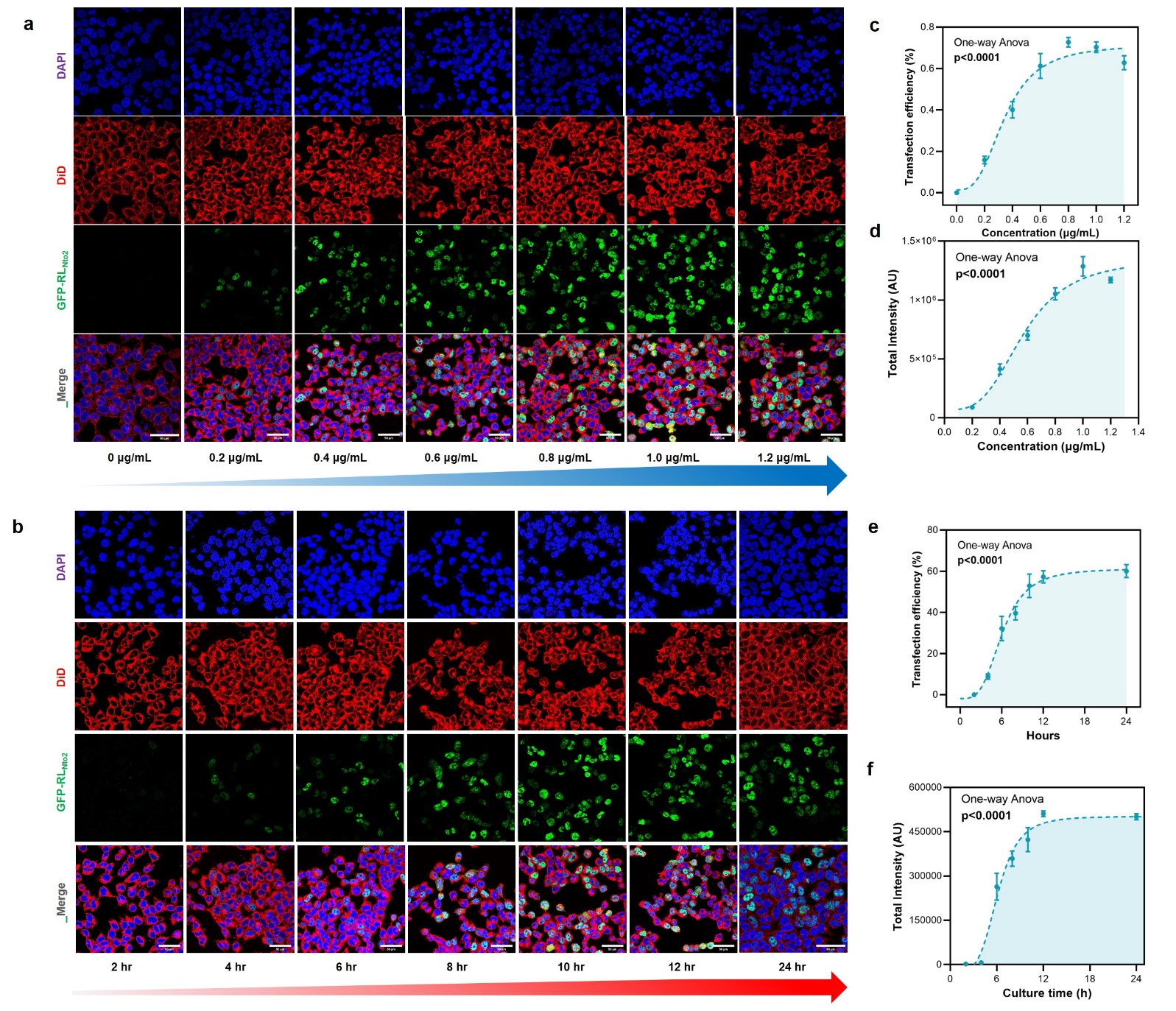


**Figure. S5** Dose-dependent and Time-scale Expression of RL_Nto2_, with different plasmid concentrations and expression durations.


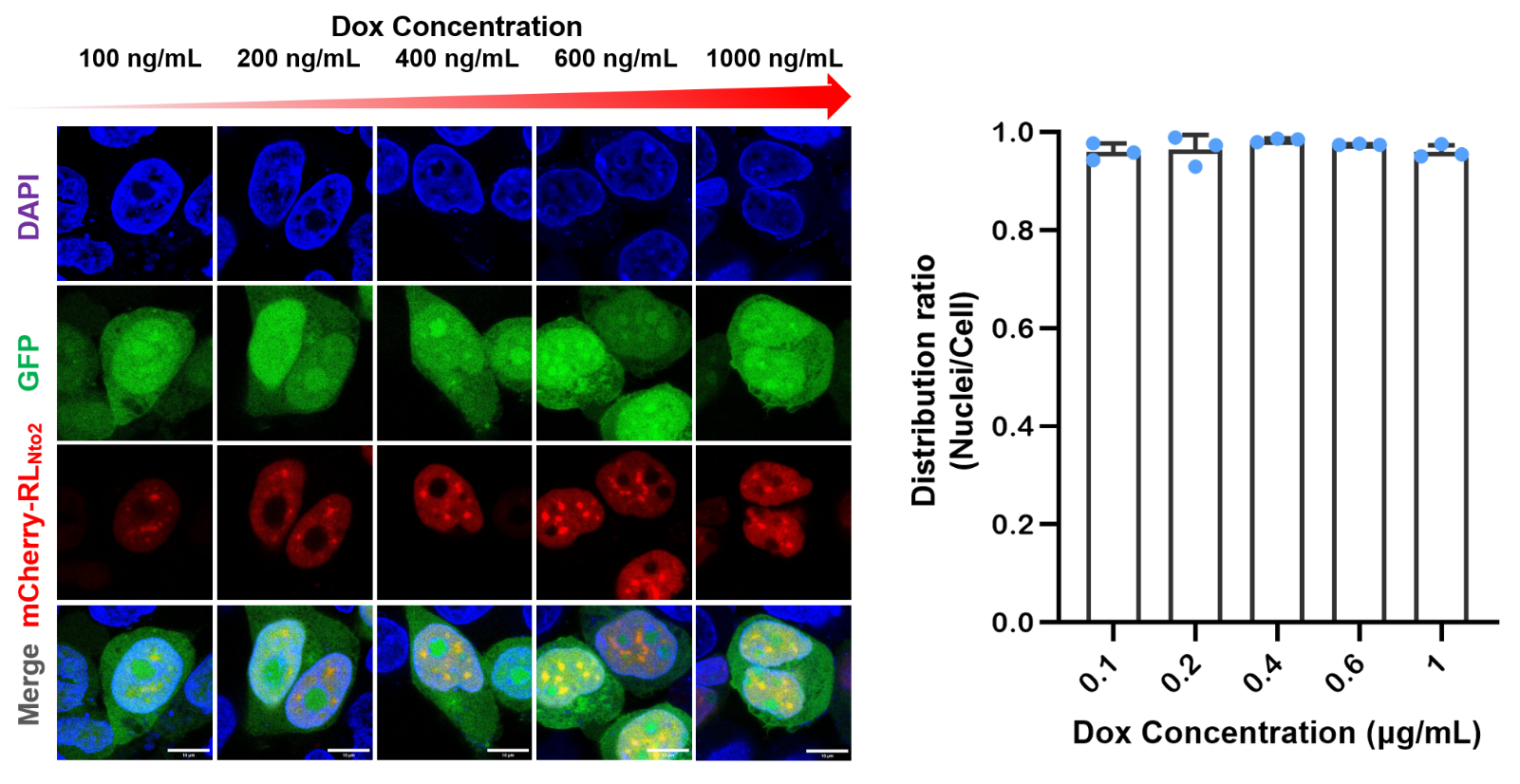


**Figure. S6** Dox-concentration-dependent expression of mCherry-RL_Nto2_ and its nuceloplasmic enrichment.
